# The Plasma Soluble Urokinase Plasminogen Activator Receptor Is Related to Disease Activity of Patients with ANCA-Associated Vasculitis

**DOI:** 10.1155/2020/7850179

**Published:** 2020-04-07

**Authors:** Fei Huang, Yueqiang Li, Ranran Xu, Anying Cheng, Yongman Lv, Qingquan Liu

**Affiliations:** ^1^Department of Nephrology, Tongji Hospital, Tongji Medical College, Huazhong University of Science and Technology, Wuhan, China; ^2^Department of Health Management Centre, Tongji Hospital, Tongji Medical College, Huazhong University of Science and Technology, Wuhan, China

## Abstract

**Objective:**

The soluble urokinase plasminogen activator receptor (suPAR) is associated with kidney diseases and is used as a prognostic factor of renal function progression. The aim of this study was to explore whether circulating suPAR was associated with antineutrophil cytoplasmic autoantibody- (ANCA-) associated vasculitis (AAV) disease activity.

**Methods:**

We evaluated 90 AAV patients with follow-up data and 35 normal controls; their plasma suPAR and C-reactive protein (CRP) levels were measured by ELISA. Associations between these levels, clinical parameters, and prognosis were analyzed.

**Results:**

Plasma suPAR levels in AAV patients were significantly higher than in healthy controls (5,920.08 ± 3,447.17 vs. 1,441.97 ± 835.04 pg/mL, *P* < 0.001). Furthermore, suPAR was significantly elevated in AAV patients in active stage compared to those in partial remissions (6,492.19 ± 3,689.48 vs. 5,031.86 ± 2,489.01 pg/mL, *P* = 0.039). Correlation analyses demonstrated that suPAR levels positively correlated with initial serum creatinine, BVAS, CRP, and procalcitonin concentration, and negatively correlated with eGFR and C3 circulating levels. In a Kaplan-Meier survival analysis, patients with plasma suPAR levels >5683.3 pg/mL showed poorer survival than patients with lower levels (log-rank, *P* = 0.001). Besides, multivariate analyses confirmed that plasma suPAR levels were an independent adverse prognostic factor for a composite outcome of end-stage renal disease (ESRD) or death, after adjusting for age and gender (HR 1.05, 95% CI = 1.01 − 1.11, *P* = 0.043). Receiver operating characteristic curves showed a suPAR cutoff value >6662.2 pg/mL for composite outcome with 68% sensitivity and 88% specificity, with an AUC = 0.82, (95% CI = 0.68 − 0.96, *P* < 0.001).

**Conclusion:**

Circulating suPAR levels might be a marker of activity correlated with disease activity in AAV patients, and, to some extent, could be a factor of poor prognosis.

## 1. Introduction

Antineutrophil cytoplasmic autoantibody- (ANCA-) associated vasculitis (AAV) is a kind of autoimmune disease characterized by pauci-immune necrotizing inflammation of the small blood vessels, with an annual incidence of 13-20/million inhabitants [1]. AAV involves multiple organ systems all over the body and seriously threatens human health; it includes granulomatosis with polyangiitis (GPA), microscopic polyangiitis (MPA), and eosinophilic granulomatosis with polyangiitis (EGPA) [2, 3]. Kidneys are the most susceptible organs; the disease is frequently manifested as a pauci-immune focal segmental necrotizing glomerulonephritis with a very rapid decline in renal function [4]. ANCAs are the serologic markers of AAV against neutrophil cytoplasmic constituents, among which myeloperoxidase (MPO) and proteinase 3 (PR3) are the two most important target antigens.

Soluble urokinase plasminogen activator receptor (suPAR) is a protein derived from the cleavage and release of the cell membrane-bound urokinase plasminogen activator receptor (uPAR), which is part of the plasminogen activation system and implicated in pathological processes of inflammation, proteolysis, tissue remodeling, and cancer metastasis [5, 6]. Several studies have demonstrated the role of suPAR as a potential biomarker in various types of cancer, cardiovascular disease, liver fibrosis, renal disorders, and systemic lupus erythematosus, as well as in rheumatic diseases where elevated suPAR levels have been related to poor prognosis [7–11]. Many kinds of cells express uPAR, including vascular smooth muscle cells, endothelial cells, monocytes, megakaryocytes, podocytes, and activated T cells [12–15]. Besides, it has been shown that uPAR is overexpressed in inflammatory cells after activation by cytokines, whereas plasma suPAR levels further increase under inflammatory or infectious conditions [16–18].

Wei et al. [19] showed that systemic levels of suPAR are significantly elevated in patients with focal segmental glomerulosclerosis (FSGS) and determined that these raised suPAR levels might be themselves causing FSGS. Moreover, Huang et al. [20] found specifically elevated urinary suPAR levels in primary FSGS patients along with a significant association between suPAR concentration and disease severity. It has been speculated that elevated suPAR could interact with podocytes by activating *β*3 integrin. Our previous studies have also revealed that plasma suPAR levels are associated with the degree of tubular atrophy/interstitial fibrosis and with the percentage of crescent formation in patients with FSGS-like lesions in IgA nephropathy (IgAN). suPAR levels may be a prognostic factor of these alterations [21]. Additionally, patients with minimal change disease and membranous nephropathy have higher plasma suPAR levels [22]. Moreover, Qin et al. [23] recently reported that plasma suPAR levels were significantly increased in patients with active lupus nephritis; besides, elevated suPAR levels were associated with some clinicopathological features related to poor prognosis. A recent study indicated independent associations between increased plasma suPAR levels and incident chronic kidney diseases (CKD) [24]. Fujimoto et al. [25] reported significant higher suPAR levels in 13 patients with MPO-ANCA-associated glomerulonephritis (ANCA-GN) and indicated that these levels might be a useful marker to assess clinical severity of ANCA-GN. Meanwhile, the role of suPAR in AAV is unclear. Whether suPAR is associated with disease activity and prognosis of AAV is not known. We speculated that suPAR might be involved in the development of AAV.

In the current study, we measured the concentration of plasma suPAR from Chinese AAV patients. Our main purpose was to explore the independent association between plasma suPAR and clinical parameters as well as its relationship with prognosis.

## 2. Materials and Methods

### 2.1. Study Population and Samples

A total of 90 patients consecutively diagnosed with AAV at the Department of Nephrology of Tongji Hospital, from September 2014 to September 2016, were enrolled in the study. Patients positive for either PR3-ANCAs or MPO-ANCAs were considered for inclusion (*n* = 146). All of them met the criteria of the 2012 Chapel Hill Consensus Conference definition for AAV [26]. Patients with secondary vasculitis (*n* = 11), diabetes mellitus (*n* = 6), tumors (*n* = 3), coexistence of other kidney diseases (*n* = 19), or other autoimmune diseases (*n* = 17) were excluded. Patients' evolution was followed until death or until the end of the follow-up period (1st September 2019). Disease control was collected from the Department of Nephrology of Tongji Hospital, including 15 patients with IgA nephropathy and 10 patients with lupus nephritis (LN). And 35 healthy participants from the Health Management Centre of Tongji Hospital were considered as normal controls.

Fasting (over 12 h) blood samples were collected from each participant and were centrifuged at 1000 g for 15 mins within 30 minutes after collection; plasma was then divided into aliquots and stored at -80°C until use. During the whole study, repeated freeze-thaw cycles of the samples were avoided.

This study was approved by the ethics committee of Tongji Hospital, Huazhong University of Science and Technology. All subjects provided informed written consent documents in accordance with the Declaration of Helsinki prior to enrollment.

### 2.2. Routine Laboratory Measurements

For all the enrolled patients, laboratory analyses information, including serum creatinine, high-sensitive C-reactive protein (CRP), erythrocyte sedimentation rate (ESR), procalcitonin (PCT), serum complement 3 (C3), and 24 h urine protein excretion levels, was derived from medical records. Serum CRP was measured using commercially enzyme-linked immunosorbent assay (ELISA) kits (Cat. no. LS-F26721, Lifespan Bio) according to the manufacturer's instructions. The estimated glomerular filtration rate (eGFR) was calculated by means of the CKD-EPI equation [27].

### 2.3. Plasma suPAR Assay

Plasma suPAR levels were tested by using a commercially available Human uPAR Quantikine ELISA kit (Catalog Number DUP00, R&D Systems, Minneapolis, MN) according to the manufacturer's instructions. The suPAR concentration from each sample was calculated using Curve expert 1.3 (Hyams DG, Starkville, Mississippi, USA). suPAR was analyzed every six months and finally was analyzed again after the collection of all samples. The average of the two measurements was taken as the final results. All assays were run in duplicate; when standard errors were over 10%, all samples were routinely reanalyzed.

### 2.4. Definitions

The composite endpoint was defined as the renal function progressed to end-stage renal disease (ESRD) or all-cause mortality, whichever occurred first. ESRD was defined as the need for renal replacement therapy (such as hemodialysis or peritoneal dialysis). The disease activity of AAV was measured at the time of sample collection by using the Birmingham Vasculitis Activity Score (BVAS) [28]. Patients at active disease stage were defined as those who had a new onset AAV or a relapse of AAV with was a new/worse disease and BVAS ≥1. “Partial remission” was determined when “after treatment, patient's disease activity BVAS score was at least 50% lower than the baseline score and absence of new manifestations” [29].

### 2.5. Immunohistochemical Staining of uPAR on Human Renal Biopsies

To observe uPAR expression in renal tissues, immunohistochemical staining of uPAR in renal biopsies from patients with AAV and normal renal tissues was performed as described previously [30]. Normal renal tissues obtained from the normal part of nephrectomized kidneys (because of renal carcinoma) were used as normal controls. In brief, renal biopsy specimens were fixed in 4% formalin and embedded in paraffin. Then, 2 *μ*m-thick tissue specimens were dewaxed and rehydrated. We performed endogenous peroxidase blocking and sequential antigen retrieval after specimens were deparaffinized. Sections were incubated with primary antibodies (uPAR, 1 : 100 dilution, Proteintech, Wuhan, China) overnight at 4°C, followed by incubation with peroxidase-conjugated secondary antibodies at 37°C for 30 min. Next, slides were counterstained with hematoxylin. uPAR expression was observed under the light microscope.

### 2.6. Differentially Expressed uPAR Analysis by Nephroseq Database

To investigate the difference of the expression uPAR gene in glomeruli, we applied the Nephroseq database (https://www.nephroseq.org/resource/main.html). The total microarray of human renal biopsy samples from 21 healthy living donors and 23 vasculitis patients. We performed unpaired *t* test for comparisons between groups and set the criterion of ∣log2 fold change (FC)∣ > 2 and two-tailed value of *P* < 0.05 as statistically significant.

### 2.7. Statistical Analysis

Data of continuous variables are expressed as mean ± s.d., nonnormally distributed continuous variables are expressed as medians and interquartile range (25th-75th), and categorical variables are shown as frequencies. Differences between the quantitative parameters of two groups were analyzed using either a *t*-test or a nonparametric test. Comparison of suPAR levels in patients repeatedly sampled at different phases of disease was done using a paired sample *t*-test. The Pearson's test was used for the correlations between suPAR and BVAS, C3, CRP, ESR, serum BUN, and urinary protein concentrations; the Spearman's test was used for the correlations between suPAR and serum creatinine levels, eGFR, PCT, and haematuria. The Kaplan-Meier curve was used to analyze outcomes, and the log-rank test was used to determine the statistical significance. Univariate and multivariate Cox regression analyses were performed for analyzing the relationship between suPAR levels and the composite outcome, with data presented as the hazard ratio (HR) and their 95% confidence interval (CI). Variables related with clinical outcomes, including initial serum creatinine and urinary protein, and age and sex were entered in the multivariate analysis. The assessment of the accuracy of plasma suPAR levels for the prediction of AAV prognosis was done by receiver operating characteristic (ROC) curves. Based on these ROC curves, the optimal cutoff values were obtained by using Youden's Index. Statistical analyses were performed with the SPSS software (SPSS, Chicago, IL), whereas the GraphPad Prism software (Graph software, San Diego, CA) was used to make graphs. Two-tailed *P* values <0.05 were considered statistically significant.

## 3. Results

### 3.1. Study Participants and Clinical Characteristics

A total of 90 patients with AAV were included in the study; 41.1% of them were male. The mean age of the study population was 53.8 years. The average concentration of plasma suPAR was 5,920.08 ± 3,447.17 pg/mL. The level of BVAS was 19.3 ± 5.8, and the initial serum creatinine concentration was 240.0 (112.0, 475.0) *μ*mol/L.  Table 1 shows the general clinical characteristics for these patients. Anti-MPO and anti-PR3 antibodies were present in 80 (88.9%) and 10 (11.1%) patients, respectively. Among the 35 healthy controls, 42.9% (15/35) were male, with an age of 54.3 ± 16.1 years. For the 15 patients with IgAN, the mean age was 36.8 ± 15.2 years, and 6 patients were male. For the 10 patients with LN, the mean age was 51.5 ± 18.9 years, and 3 patients were male.

### 3.2. Plasma suPAR Levels in AAV Patients and Controls

**Figure 1:**
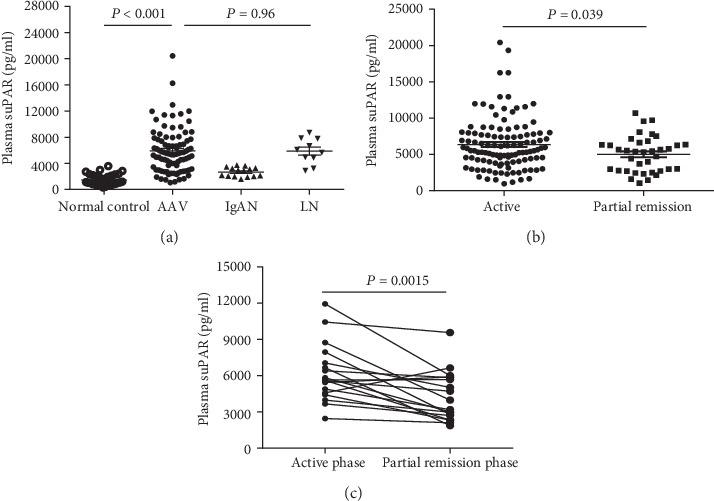
Plasma levels of suPAR in different groups. (a) Plasma levels of suPAR in antineutrophil cytoplasmic autoantibody-associated vasculitis (AAV) patients, patients with IgAN and LN, and normal controls. (b) Plasma levels of suPAR in AAV patients at active (*n* = 57) and partial remission stages (*n* = 33). (c) Changes of plasma suPAR levels in 18 AAV patients after the analysis of sequential plasma samples. These 18 AAV patients mean BVAS at the two measurement points were 15.56 and 6.06, and the mean interval between active stage and partial remission stage was 77.3 days.

Plasma samples of patients with AAV, IgAN, LN, and healthy controls were collected. As shown in Figure 1(a), mean levels of plasma suPAR levels in patients with AAV were higher compared with healthy controls (5,920.08 ± 3,447.17 vs. 1,441.97 ± 835.04 pg/mL, *P* < 0.001). Subsequent analysis revealed that AAV patients in active stage had higher suPAR levels than patients in partial remission (6,492.19 ± 3,689.48 vs. 5,031.86 ± 2,489.01 pg/mL, *P* = 0.039) (Figure 1(b)). But there was no significant difference in the plasma suPAR levels between AAV patients and LN patients (5,920.08 ± 3,447.17 vs. 5,870.38 ± 1,953.83 pg/mL, *P* = 0.96). Moreover, immunohistochemical staining showed increased expression of uPAR in AAV patients than in healthy controls both in glomeruli and tubules (Supplemental Figure 1). By analyzing the publicly accessible Nephroseq dataset (https://www.nephroseq.org/resource/main.html), we found that the expression of the glomerular uPAR protein in vasculitis patients was significantly greater than that in healthy controls (Supplemental Figure 2) [31]. In addition, after excluding patients with PCT above 0.5 ng/mL, AAV patients with PCT levels below 0.5 ng/mL still had significant higher suPAR levels than healthy controls (Supplemental Figure 3, 5,393.60 ± 1,086.19 vs. 1,441.97 ± 835.04 pg/mL, *P* < 0.001).

Furthermore, among the 57 patients who were initially diagnosed as AAV with renal damage, 30 of them returned to the outpatient clinic of our hospital two months after discharge, and 18 of them reached partial remission based on clinical symptoms and the BVAS. We compared plasma suPAR concentrations of these 18 AAV patients who were sampled repeatedly in different phases of disease (active stage and partial remission). A significant decrease in plasma suPAR levels was found after partial disease remission (6,186.51 ± 2,379.69 vs. 4,260.78 ± 2,055.22 pg/mL, *P* = 0.0015): 16 of these 18 patients had lower levels of plasma suPAR after reaching the partial remission stage than in the active disease stage, and only 2 patients had increased levels of plasma suPAR in the partial remission stage (Figure 1(c)). These 18 patients did not differ significantly from the remaining 39 initially diagnosed patients in terms of treatment plan (glucocorticoids + cyclophosphamides) or baseline plasma suPAR levels (6186 ± 560.7 vs. 7043 ± 624.2 pg/mL, *P* = 0.380).

### 3.3. Relationships between Plasma suPAR Levels and Clinical Parameters in AAV Patients

**Figure 2:**
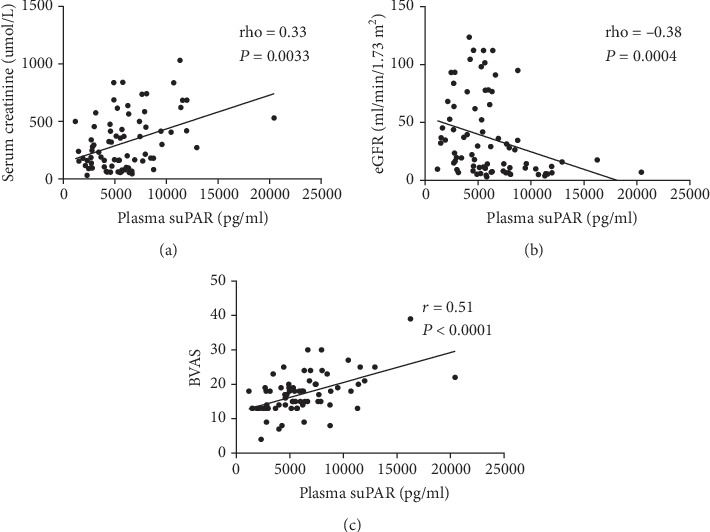
Correlation of plasma suPAR levels with (a) serum creatinine, (b) eGFR, and (c) Birmingham Vasculitis Activity Score (BVAS).

**Figure 3:**
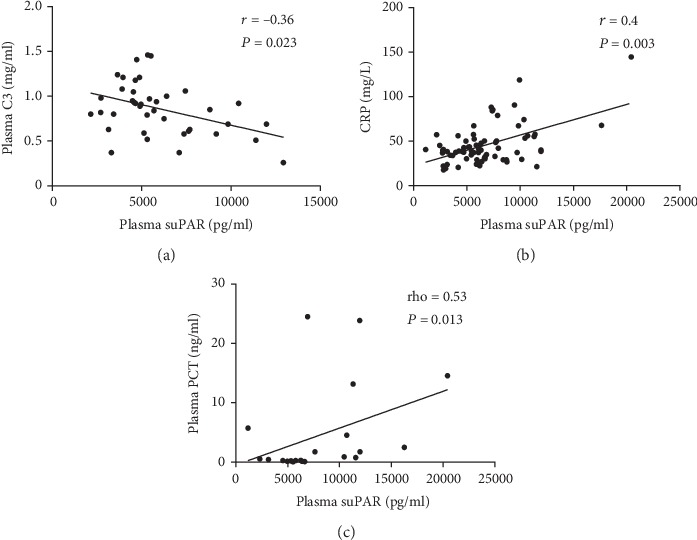
Correlation of plasma suPAR levels with (a) plasma C3 levels (*n* = 40), (b) plasma CRP levels (*n* = 74), and (c) plasma PCT levels (*n* = 21).

We analyzed the association between suPAR expression levels and clinical parameters showing disease activity in AAV patients (Figures 2 and 3). Significant negative correlations were observed between plasma levels of suPAR and serum levels of C3 and eGFR (*r* = −0.36, *P* = 0.023 and rho = −0.38, *P* = 0.0004, respectively). In addition, significant positive correlations were found between the plasma levels of suPAR and serum creatinine levels, serum BUN, BVAS, CRP, and PCT (rho = 0.33, *P* = 0.0033; *r* = 0.32, *P* = 0.007; *r* = 0.51, *P* < 0.001; *r* = 0.40, *P* = 0.0029; and rho = 0.53, *P* = 0.013, respectively). In contrast, plasma suPAR levels failed to correlate with ESR, urinary protein concentrations, or haematuria (*r* = 0.037, *P* = 0.823; *r* = −0.251, *P* = 0.086; and rho = 0.177, *P* = 0.301, respectively).

### 3.4. Relationship between Plasma suPAR Levels and Outcomes of AAV Patients

**Figure 4:**
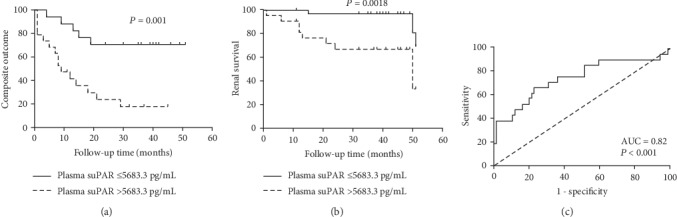
Association between plasma levels of suPAR and prognosis of patients with AAV. (a) Kaplan-Meier survival curves showed associations with composite outcomes (death or ESRD) according to suPAR status. (b) Kaplan-Meier survival curves showed associations with ESRD according to suPAR status. (c) Receiver operating characteristic (ROC) analysis of suPAR in patients with AAV.

The median length of follow-up of patients with AAV was 22 (range 1-51) months. During the follow-up period, 18 patients died, 11 patients progressed to ESRD, and 29 patients developed a composite endpoint. We observed significant higher plasma suPAR levels in AAV patients who developed a composite endpoint than those without it (7,991.30 ± 810.68 vs. 5,136.39 ± 337.10 pg/mL, *P* < 0.001). After dividing patients into two equal size groups, based on plasma suPAR levels, we used the Kaplan-Meier survival curve analysis to assess the relationship of plasma suPAR levels and the prognosis of patients (Figure 4). The time to composite outcome or ESRD of patients with plasma suPAR levels >5,683.3 pg/mL was significantly shorter than patients with lower suPAR levels (*P* = 0.001, *P* = 0.0018, respectively). What is more, the associations between serum suPAR and the composite outcome or ESRD remained significant in the Kaplan-Meier survival analysis after excluding patients with a follow-up fewer than 3 months (Supplemental Figure 4, *P* = 0.0075, *P* = 0.045, respectively).

Table 2 presents the HRs and 95% CIs for renal prognosis in patients with AAV according to the status of their plasma suPAR levels. A univariate Cox regression analysis indicated a role of plasma suPAR levels as a risk factor for a composite outcome, with a HR of 1.07 (95% CI = 1.03 − 1.12, *P* = 0.002). Adjusting a multivariate Cox regression analysis for age and sex only did not affect the significance of plasma suPAR levels in the endpoint outcome (HR = 1.05, 95% CI = 1.01 − 1.11, *P* = 0.043). After an adjustment including age, gender, and serum creatinine and urinary protein concentrations, plasma suPAR levels failed to be associated with a composite outcome (HR = 1.02, 95% CI = 0.96 − 1.08, *P* = 0.563). After excluding patients with a follow-up fewer than 3 months, plasma suPAR was found to be associated with composite outcome in the univariate COX regression (Supplemental Table 1, HR = 1.07, 95% CI = 1.03 − 1.12, *P* = 0.002).

To verify the prediction efficiency of plasma suPAR for composite outcomes, a ROC curve analysis was generated for patients with different outcomes (area under the curve [AUC] = 0.82, 95% CI = 0.68 − 0.96, *P* < 0.001) (Figure 4(c)). Results calculated by using Youden's index showed a cutoff value for suPAR concentration of 6,662.2 pg/mL with a sensitivity of 68% and a specificity of 88%.

## 4. Discussion

suPAR, a protein derived from cleavage and release from the cell membrane-bound uPAR, has been reported to be associated with a broad range of diseases [7–11]. In recent years, increasing evidences have revealed a potential bioactive role of serum suPAR in FSGS etiology. Moreover, it has been proposed as a useful tool to predict renal function progression. To our knowledge, data on serum suPAR levels in patients with AAV is sparse, so we assessed the association between serum suPAR levels and both disease activity and prognosis of AAV patients.

In the current study, we found elevated plasma levels of suPAR in 90 patients with AAV in relation to those seen in healthy controls. Consistently, this was paralleled by an increase in the expression of uPAR in renal biopsies in AAV patients. Prior reports had shown that there were remarkably higher suPAR levels in 13 patients with MPO-ANCA-associated glomerulonephritis compared with healthy controls [24], as well as significantly higher suPAR levels in 5 ANCA-GN patients than in non-ANCA-GN patients [32]. These two reports briefly described the relationship of suPAR levels and disease activity using limited sample sizes and did not show any potential prognosis role of suPAR. Our findings aligned well with their observations in a relatively large sample cohort; furthermore, we reported lower suPAR levels in AAV patients when moving from active disease to partial remission, reflecting a relationship of suPAR concentration with the inflammatory process in AAV.

Similar to Fujimoto et al.'s results [25], we also detected that the suPAR levels positively correlated with CRP and inversely correlated with eGFR levels. In addition, we demonstrated that there is an association between the concentration of suPAR with other clinical parameters in AAV, including BVAS, PCT, and C3, further revealing a close relationship between suPAR levels and disease activity. It is worth mentioning that there is an inverse correlation between suPAR levels and kidney function, which was reported not only for AAV but also for chronic kidney disease and other glomerular diseases [32, 33]. Elevated suPAR levels might be a result of decreased eGFR as for suPAR, whose major fragment has a molecular weight of 22 kDa, may be easier to pass through the glomerular filtration barrier [10]. This phenomenon should not be simply attributed to renal clearance, as it might also be related to the underlying pathogenesis that initiates kidney disease. Plasma suPAR has been postulated to lead to acute proteinuric kidney disease, specifically to FSGS [19, 34]. Our previous studies have also reported associations between suPAR levels and proteinuria in FSGS-like lesions in IgA nephropathy [21]. In contrast, suPAR did not seem to correlate with proteinuria in AAV, either in this or in Fujimoto's research. Because AAV has a rapidly progressive clinical course, we speculate that urinary abnormalities may also result from renal scarring and may not necessarily reflect any disease activity. In fact, recent in vitro and in vivo studies have produced conflicting results on the effect of suPAR on podocyte injury and proteinuria [35, 36]; therefore, unless further clinical evidence is provided, the role of suPAR as a possible causative factor in FSGS should be seriously considered. Taken together, the above results indicate that plasma suPAR levels are associated with renal involvement and disease activity in patients with AAV.

Accumulating animal and clinical studies have indicated that the alternative complement pathway activation might be involved in the development of AAV [37–40]. As part of the alternative complement pathway, C3 were found to correlate with suPAR negatively in our study. The activation of uPAR/suPAR could activate plasminogen and generate plasmin, which cleaves both C3 and C5 to C3a and C5a [41]. This interaction might be a reason to explain the correlation between suPAR and C3 levels. We also detected strong correlations between plasma suPAR levels and inflammation markers, such as CRP and PCT. Studies have shown a direct chemotactic property of suPAR that facilitates recruitment of inflammatory cells [42, 43], among which ANCA-activated monocytes release proinflammatory cytokines like interleukin-6 (IL-6) [37]. These cytokines induced by the interaction between suPAR and inflammatory cells therefore might stimulate synthesis and subsequent release of CRP [44]. PCT has been proposed as a specific biomarker of severe systemic bacterial, fungal infections, and sepsis. To date, a few studies investigated AAV patients for PCT levels. Studies showed that AAV patients without evidence of infection could have elevated serum PCT levels, but other reports possess opposite results that PCT was not significantly increased in disease activity in various autoimmune diseases including GPA [45–47], so further investigations are needed to discuss this controversial issue. Briefly, plasma suPAR could serve as a factor reflecting an inflammatory condition, but further studies are still needed to clarify whether increased suPAR is caused by overactivation of inflammation in patients with AAV or if it actually exerts a proinflammatory role.

Moreover, we observed, for the first time, a strong positive association between plasma suPAR levels and composite outcome of AAV patients after an adjustment of both age and sex. But this association disappeared after considering the initial serum creatinine concentration, which might be explained by the close correlation between suPAR levels and renal function. Therefore, we propose that plasma suPAR might participate in pathogenesis of AAV but does not accelerate disease progression. Nevertheless, ROC curve analyses showed that suPAR has a promising prognostic value (AUC = 0.82) for the composite outcome of AAV, indicating a relationship between elevated suPAR levels and high risk of poor outcomes and revealing a potential pathophysiological significance for suPAR. We are interested in further studying this system to clarify if suPAR could be involved in the pathogenesis mechanism of AAV.

Our study also has several limitations that merit consideration. First, although most of the results were statistically significant, the sample size of the study was small. Second, we did not analyze the relationship between plasma suPAR levels and renal pathological parameters of renal tissue in AAV patients. Third, there might be some disease assessment biases caused by the differences among the treating physician's knowledge about the ANCA status. Therefore, additional prospective studies are required to substantiate the role of plasma suPAR levels in predicting AAV patient outcomes.

## 5. Conclusions

In conclusion, our current study found that plasma suPAR levels were significantly correlated with disease activity and could be a factor of poor prognosis. Additional studies are required to investigate whether suPAR plays a role in AAV disease progression.

## Figures and Tables

**Table 1:** Clinical parameters of patients with AAV.

Characteristic	Values
Number of patients	90
Age (mean ± s.d.)	53.8 ± 18.3
Gender (male/female)	37/53
MPO-ANCA/PR3-ANCA	80/10
Initial serum creatinine (*μ*mol/L) (median, IQR)	240.0 (112.0, 475.0)
eGFR (mL/min/1.73 m^2^)^a^ (median, IQR)	22.9 (9.57, 64.23)
Urinary protein (g/24 h) (mean ± s.d.)	1.69 ± 1.58
Dialysis-dependent at presentation	17 (18.9%)
CRP (mg/L) (*n* = 74)	40.3 ± 24.1
ESR (mm/1 h) (*n* = 49)	55.0 ± 40.8
PCT (ng/mL) (median, IQR) (*n* = 21)	0.82 (0.16, 5.43)
C3 (g/L) (*n* = 40)	0.88 ± 0.29
MPO (RU/mL) (median, IQR)	159.7 (98.8, 235.7)
BVAS (mean ± s.d.)	19.3 ± 5.8

^a^eGFR (mL/min per 1.73 m^2^) = 175 × (plasma creatinine)^−1.234^ × age^−0.179^ × 0.79 (if female). Abbreviations: AAV: antineutrophil cytoplasmic antibody-associated vasculitis; BVAS: Birmingham Vasculitis Activity Scores; eGFR: estimated glomerular filtration rate; ESR: erythrocyte sedimentation rate; IQR: interquartile range; s.d.: standard deviation; C3: complement 3; MPO: myeloperoxidase; PR3: proteinase 3; ANCA: antineutrophil cytoplasmic autoantibody; CRP: C-reactive protein; PCT: procalcitonin.

**Table 2:** Multivariate analysis of composite outcome in patients with AAV.

	Univariate		Multivariate^∗^		Multivariate^†^	
	HR (95% CI)	*P* value	HR (95% CI)	*P* value	HR (95% CI)	*P* value
Plasma suPAR levels (per 500 pg/mL increase)	1.07 (1.03-1.12)	0.002	1.05 (1.01-1.11)	0.043	1.02 (0.96-1.08)	0.563
Age (year)^§^	1.04 (1.01-1.08)	0.017	1.03 (0.99-1.07)	0.128	1.05 (1.01-1.10)	0.025
Gender (male vs. female)	1.02 (0.48-2.19)	0.957	0.98 (0.46-2.11)	0.961	0.74 (0.30-1.83)	0.515
Initial serum creatinine (per mg/dL)	1.17 (1.06-1.30)	0.003	—	—	1.22 (1.06-1.41)	0.006
Urinary protein (per g/24 h)	0.93 (0.66-1.30)	0.699	—	—	1.03 (0.71-1.49)	0.879

^∗^Adjusted for age, gender; ^†^adjusted for age, gender, initial serum creatinine, and urinary protein; ^§^per 1 unit increase. Abbreviations: AAV: antineutrophil cytoplasmic antibody-associated vasculitis; HR: hazard ratio; CI: confidence interval.

## Data Availability

The data that support the findings of this study are available on request from the corresponding author Qingquan Liu. The data have not been made publicly available because they contain information that could compromise the privacy or consent of the study participants.

## Supplementary Materials

Supplementary Materials Supplemental Figure 1: immunohistochemistry staining for uPAR in glomeruli and tubules of renal specimens from normal controls and AAV patients. Supplemental Figure 2: analysis of uPAR gene expression in glomeruli of vasculitis patients. The publicly accessible Nephroseq dataset was used for the expression analysis of glomerular uPAR gene. The histogram showed significantly greater uPAR gene expression in vasculitis patients vs. healthy controls (*t*-test). Supplemental Figure 3: plasma levels of suPAR in AAV patients with PCT levels below 0.5 ng/mL and normal controls. Supplemental Figure 4: association between plasma levels of suPAR and prognosis in AAV patients with a follow-up period of 3-51 months. (a) Kaplan-Meier survival curves showed associations with composite outcomes (death or ESRD) according to suPAR status. (b) Kaplan-Meier survival curves showed associations with ESRD according to suPAR status. Supplemental Table 1: multivariate analysis of composite outcome in AAV patients with a follow-up period of 3-51 months.
