# Supplementary material for: The Plasma Soluble Urokinase Plasminogen Activator Receptor Is Related to Disease Activity of Patients with ANCA-Associated Vasculitis
Source: Mediators Inflamm. 2020 Apr 7;2020:7850179. doi: 10.1155/2020/7850179 (PMC7166284; doi:10.1155/2020/7850179)
Supplement: Supplementary Materials — Supplemental Figure 1: immunohistochemistry staining for uPAR in glomeruli and tubules of renal specimens from normal controls and AAV patients. Supplemental Figure 2: analysis of uPAR gene expression in glomeruli of vasculitis patients. The publicly accessible Nephroseq dataset was used for the expression analysis of glomerular uPAR gene. The histogram showed significantly greater uPAR gene expression in vasculitis patients vs. healthy controls (t-test). Supplemental Figure 3: plasma levels of suPAR in AAV patients with PCT levels below 0.5 ng/mL and normal controls. Supplemental Figure 4: association between plasma levels of suPAR and prognosis in AAV patients with a follow-up period of 3-51 months. (a) Kaplan-Meier survival curves showed associations with composite outcomes (death or ESRD) according to suPAR status. (b) Kaplan-Meier survival curves showed associations with ESRD according to suPAR status. Supplemental Table 1: multivariate analysis of composite outcome in AAV patients with a follow-up period of 3-51 months. [file 7850179.f1.pdf]

Supplementary Materials

Supplemental figure 1. Immunohistochemistry staining for uPAR in glomeruli and tubules of renal specimens from normal controls and AAV patients.

Supplemental figure 2. Analysis of uPAR gene expression in glomeruli of vasculitis patients. The publicly accessible Nephroseq dataset was used for the expression analysis of glomerular uPAR gene. The histogram showed significantly greater of uPAR gene expression in vasculitis patients vs healthy controls (t-test).

Supplemental figure 3. Plasma levels of suPAR in AAV patients with PCT levels below 0.5 ng/mL and normal controls.

Supplemental figure 4. Association between plasma levels of suPAR and prognosis in AAV patients with a follow-up period of 3-51 months. (A) Kaplan-Meier survival curves showed associations with composite outcomes (death or ESRD) according to suPAR status. (B) Kaplan-Meier survival curves showed associations with ESRD according to suPAR status.

Supplemental table 1. Multivariate analysis of composite outcome in AAV patients with a follow-up period of 3-51 months.

Supplemental figure 1

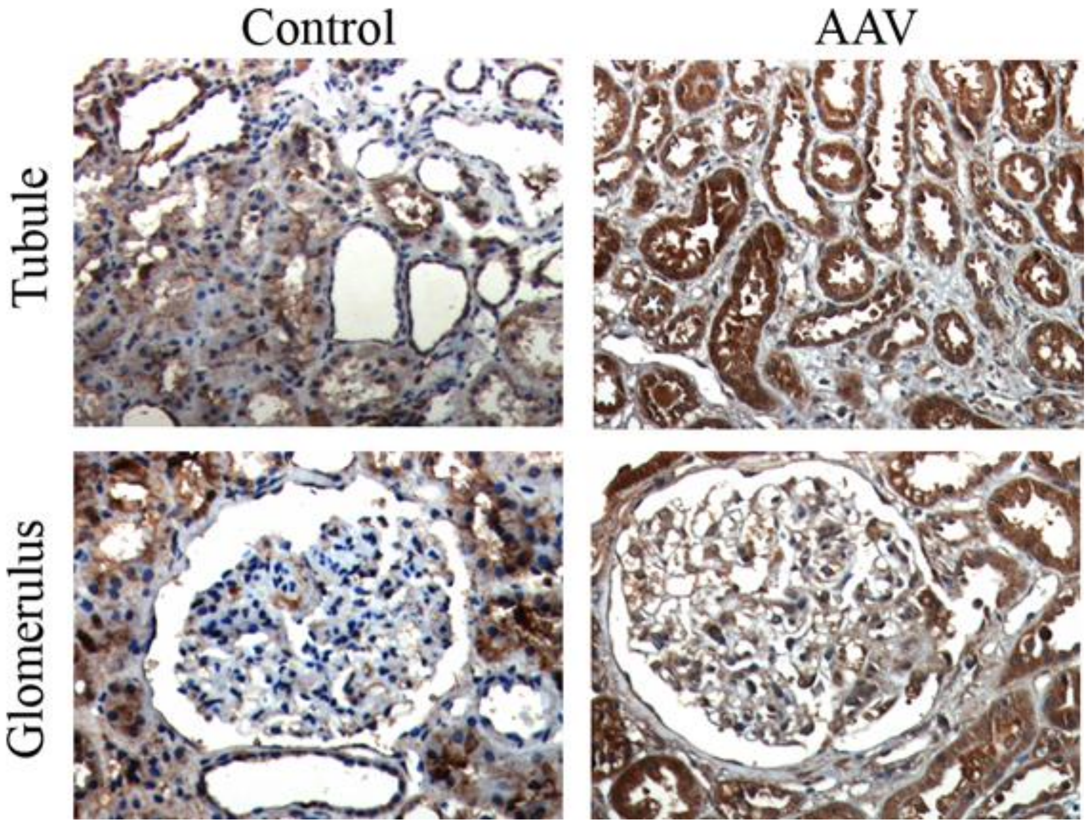

Immunohistochemistry staining for uPAR in glomeruli and tubules of renal specimens from normal controls and AAV patients.

Supplemental figure 2

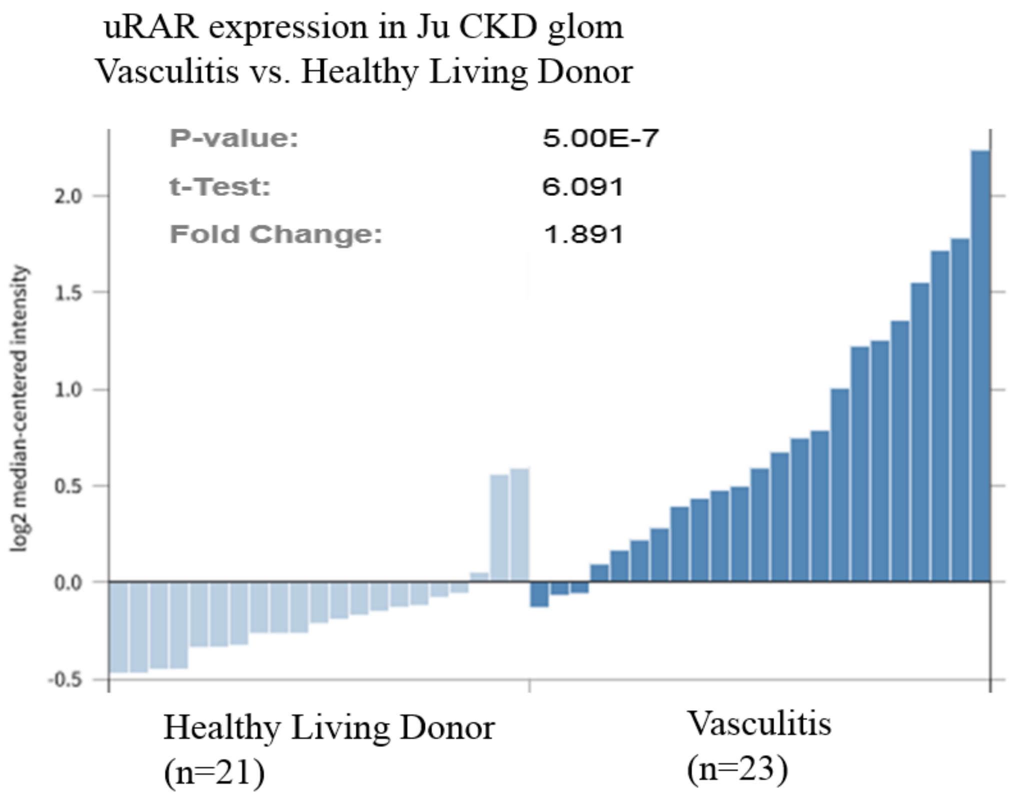

Analysis of uPAR gene expression in glomeruli of vasculitis patients. The publicly accessible Nephroseq dataset was used for the expression analysis of glomerular uPAR gene. The histogram showed significantly greater of uPAR gene expression in vasculitis patients vs healthy controls (t-test).

Supplemental figure 3

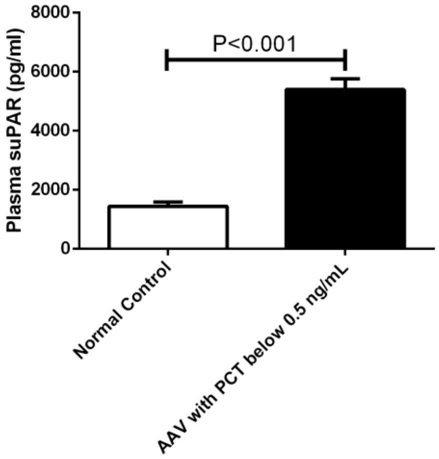

Plasma levels of suPAR in AAV patients with PCT levels below 0.5 ng/mL and normal controls.

Supplemental figure 4

A

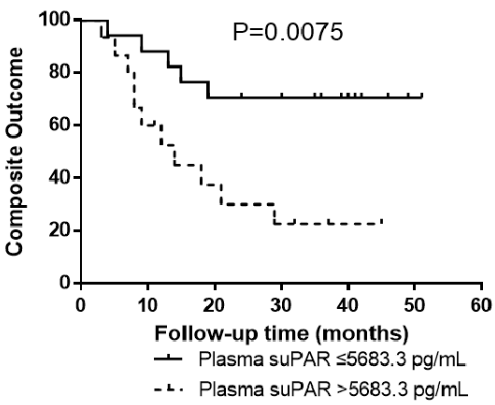

B

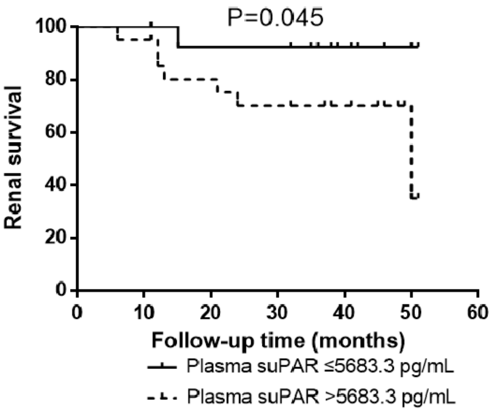

Association between plasma levels of suPAR and prognosis in AAV patients with a follow-up period of 3-51 months. (A) Kaplan-Meier survival curves showed associations with composite outcomes (death or ESRD) according to suPAR status. (B) Kaplan-Meier survival curves showed associations with ESRD according to suPAR status.

Supplemental table 1. Multivariate analysis of composite outcome in AAV patients with a follow-up period of 3-51 months.

	Univariate		Multivariate*		Multivariate†	
	HR (95% CI)	P value	HR (95% CI)	P value	HR (95% CI)	P value
Plasma suPAR levels (per 500 pg/mL increase)	1.07(1.03-1.12)	0.002	1.06(0.97-1.16)	0.158	1.00(0.92-1.10)	0.935
Age, yr§	1.10(1.04-1.16)	0.001	1.07(1.02-1.14)	0.012	1.12(1.04-1.20)	0.002
Gender (male vs. female)	0.69(0.27-1.80)	0.453	0.64(0.24-1.74)	0.385	0.32(0.09-1.14)	0.079

Initial serum creatinine (per mg/dl)	1.16(1.04-1.29)	0.010	-	-	1.43(1.11-1.84)	0.006
Urinary protein (per g/24 h)	0.91(0.61-1.34)	0.624	-	-	1.98(0.61-1.57)	0.934
